# Successful rescue of PD-1 inhibitor-induced TEN by vincristine combination therapy: a case report and literature review

**DOI:** 10.3389/fonc.2025.1621494

**Published:** 2025-09-10

**Authors:** Hui-Yi Huang, Jia-Qi Liu, Yun-Yun Zeng, Zhi-Hao Huang, Shan Su

**Affiliations:** Department of Oncology, Guangzhou Chest Hospital, Guangzhou, China

**Keywords:** vincristine (VCR), toxic epidermal necrolysis (TEN), immune-related adverse events (IRAE), PD-1 inhibitor, immune check point inhibitor (ICI)

## Abstract

With the growing use of immune checkpoint inhibitors (ICIs) in the management of various solid malignancies, immune-related adverse events (irAEs) are increasingly recognized. Toxic epidermal necrolysis (TEN), though rare, represents a severe and potentially fatal cutaneous irAE with a high mortality rate. Owing to its low incidence and limited clinical data, a standardized treatment approach for ICI-induced TEN has not been established. We present a patient who developed TEN following the administration of a programmed cell death-1 inhibitor. The patient was successfully managed with a combination of vincristine, intravenous immunoglobulin, and glucocorticoids at the Department of Oncology, Guangzhou Chest Hospital.

## Introduction

Toxic epidermal necrolysis (TEN) is a rare, life-threatening dermatological condition, most commonly induced by medications, which accounts for approximately 85% of patients; it is characterized by widespread blistering, epidermal detachment, extensive mucosal involvement, and systemic dysfunction, with epidermal sloughing affecting > 30% of the total body surface area (1). TEN has been reported in association with the use of immune checkpoint inhibitors (ICIs) (2–4). ICI-induced TEN may initially manifest as a refractory maculopapular rash, which can progress to generalized erythema and petechiae, followed by the development of blisters, maculopapules, or hematomas, and extensive necrotic exfoliation of the skin and mucous membranes. This progression is often accompanied by fever and multisystem involvement (5–7). Notably, the mortality rate of TEN associated with ICIs exceeds 55%, significantly higher than that observed in TEN of other etiologies (8). Considering the high fatality associated with ICI-induced TEN, early identification and the prompt initiation of effective treatment are critical to improving clinical outcomes. The SCORTEN scoring system is widely used to assess disease severity within 24 h of hospital admission (9). It provides prognostic stratification, with higher scores correlating with increased mortality risk. Due to the low prevalence of TEN and the limited availability of robust clinical data, no standardized treatment guidelines have been established. According to the Expert Consensus on the Diagnosis and Treatment of Stevens-Johnson Syndrome/Toxic Epidermal Necrolysis, empiric management includes systemic glucocorticoids, cyclosporine, and other immunosuppressants, alongside meticulous wound care (10). We present a case of TEN induced by carrelizumab, which was successfully treated with a combination of vincristine (VCR), intravenous immunoglobulin, and systemic glucocorticoids.

## Case report

### Patient information

A 51-year-old male was diagnosed with hypopharyngeal carcinoma in December 2020. On January 22, 2021, he commenced anti-tumor therapy with paclitaxel, cisplatin, and carrelizumab at the Sun Yat-sen University Cancer Prevention and Treatment Centre. On January 31, 2021, the patient developed a fever, with a maximum recorded temperature of 38.5°C, accompanied by chills. The symptoms was persistence with supportive treatment.

On February 3, 2021, he developed a scattered maculopapular rash. Anti-tuberculosis therapy was discontinued, and anti-allergic treatment (oral Cetirizine 10 mg daily)was initiated; however, the rash progressively worsened and was accompanied by pruritus, exertional dyspnea, and persistent high-grade fever.

On February 4, 2021, the patient was admitted to our hospital. He was alert but appeared lethargic, with a reduced appetite.

### Past medical history

The patient was diagnosed with tuberculosis in September 2020 and was receiving anti-tuberculosis therapy. No known drug allergies were reported.

### Admission signs

Temperature, 40°C; pulse, 128 beats per min; blood pressure, 120/76 mmHg. The patient was alert and oriented. Physical examination revealed diffuse erythema with scattered blisters. Pulmonary auscultation was unremarkable.

### Diagnosis and treatment

On the first day of admission, the patient was initiated on supportive treatments and empirical therapy. The supportive treatments comprised: single-room isolation, oxygen supplementation, hepatoprotective therapy, myocardial protection, gastric mucosal protection and nutritional supplementation. The empirical therapy consisted of anti-allergic treatment (intravenous calcium gluconate 1 g daily, oral loratadine 10 mg nightly, and intramuscular diphenhydramine 20 mg daily), antimicrobial treatment ( intravenous imipenem 1 g every 8 hours and moxifloxacin 0.4 g daily), and prophylactic antiviral treatment (intravenous acyclovir 0.5 g every 8 hours). The SCORTEN score on admission was 4.

On the second day, the patient continued to experience recurrent fevers (Tmax 38.1 °C). Erythema progressed diffusely, involving the entire body including the perineal region, accompanied by a burning sensation (numeric rating scale [NRS] pain score 5). The blisters increased in size, coalesced, and some ruptured spontaneously. The patient reported severe oropharyngeal pain. Examination revealed significant mucosal erythema and edema, although no ulceration or active bleeding was observed. Considering the clinical presentation and treatment history, a diagnosis of TEN secondary to carrelizumab (PD - 1 inhibitor) was suspected. Accordingly, treatment was escalated to include high-dose intravenous methylprednisolone (500 mg daily), human immunoglobulin (20 g daily), and sodium thiosulfate.

On the third day, the patient exhibited progressive fever with Tmax 38.6°C. The burning sensation intensified, with an NRS pain score of 6. To suppress immune activity further, VCR (1 mg infused over 4 h) was administered. From the fourth day onward, his body temperature returned to normal, the cutaneous burning sensation resolved, no new rashes emerged, and oropharyngeal pain exhibited significant improvement.

**Figure 1:**
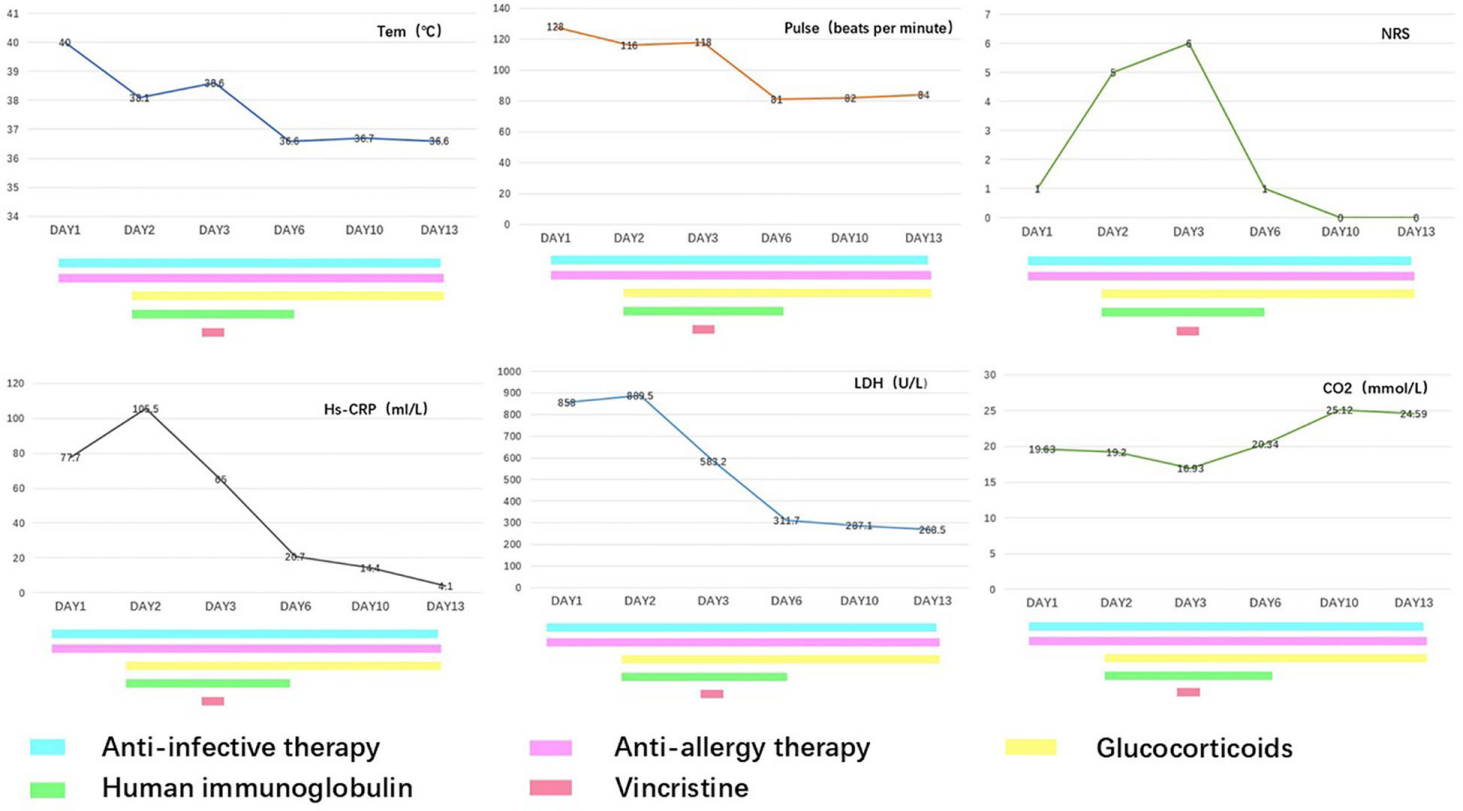
Time course of illness and inflammatory markers.

**Figure 2:**
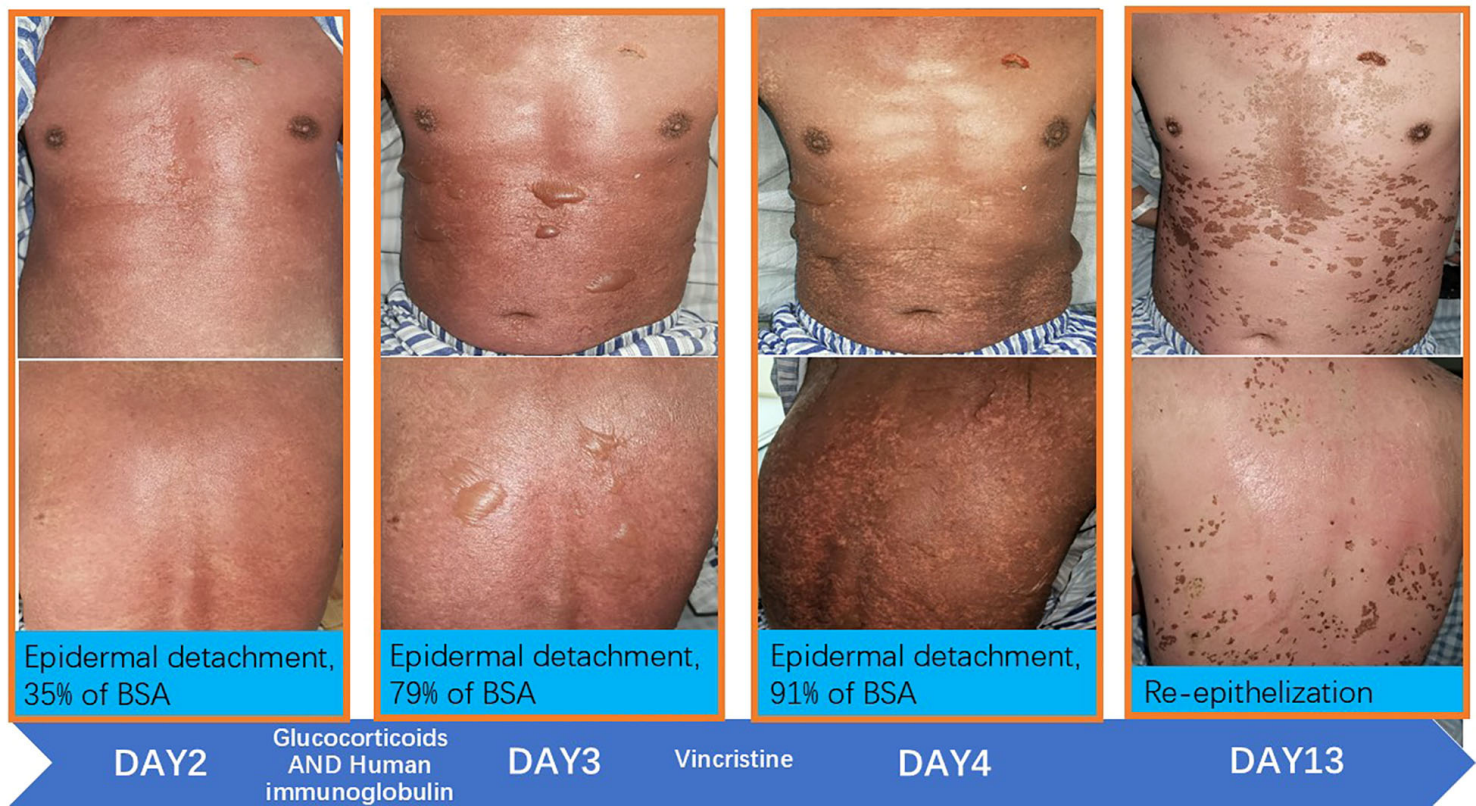
Skin condition.

During hospitalization, the therapeutic regimen included high-dose intravenous methylprednisolone (500 mg daily for 3 days, tapered to 60 mg daily for the subsequent 5 days), intravenous immunoglobulin (20 g daily for 5 days), and VCR (1 mg infused over 4 h). For local skincare, large blisters were aspirated for drainage, with efforts made to preserve the blister roof as a natural biological dressing. Potassium permanganate wet dressings were applied to areas with ruptured blisters to prevent secondary infection. Oral discomfort was managed with a compounded mouthwash consisting of lidocaine (0.2 g), gentamicin (240,000 IU), vitamin B12 (3 mg), dexamethasone (20 mg), and smectite powder (9 g). Serial laboratory data are presented in **Figure 1**, and photographic documentation of the cutaneous lesions on hospital day 2, day 3, day 4, and day 13 is provided in **Figure 2**.

On February 18, 2021, the patient was discharged in a significantly improved condition. Oral glucocorticoid therapy was continued on an outpatient basis and gradually tapered to discontinuation. Considering the severity of the adverse reaction, PD - 1 inhibitor therapy was permanently discontinued. Subsequently, the patient received two additional cycles of chemotherapy with paclitaxel and cisplatin, followed by radical radiotherapy for laryngeal cancer (exact dosage not documented). No dermatological complications or other adverse effects were noted during or after these treatments. The patient has been followed up regularly, with no evidence of tumor recurrence or metastasis to date.

## Discussion

TEN represent a spectrum of rare but severe delayed-type hypersensitivity reactions, characterized by extensive necrosis and detachment of the epidermis and mucosal epithelium. Most cases are drug-induced or associated with infections. ICI-induced TEN represents a rare but potentially life-threatening irAE. As the use of ICIs becomes more widespread, the incidence of ICI-associated TEN has correspondingly increased. The mortality rate associated with ICI-related TEN was reported to be 47.8 - 70%, significantly higher than those observed in TEN caused by other etiologies (8, 11). In this case, the SCORTEN score was 4, corresponding to an estimated mortality rate of approximately 58.3%. We successfully treated ICI-induced TEN with a combination regimen consisting of vincristine (VCR), glucocorticoids, and intravenous immunoglobulins (IVIG), achieving complete remission with a relatively short hospitalization period.

Due to the low prevalence of TEN and limited availability of high-quality clinical data, both domestic and international guidelines/consensus acknowledge the absence of robust, evidence-based protocols for standardized treatment. Based on the immunopathogenesis of TEN, systemic glucocorticoids and intravenous immunoglobulin are commonly used as first-line therapies. Emerging evidence from recent studies reveals poor outcomes in first-line treatment of ICI-induced TEN, demonstrating a high mortality rate of 55.3% (8) and prolonged median hospitalization duration of 32 days (2). These concerning findings underscore the critical unmet need for more effective therapeutic interventions in this life-threatening condition. Current additional treatment options may include cyclosporine, tumor necrosis factor-alpha (TNF-α) inhibitors, tocilizumab, plasma exchange, and cyclophosphamide (9, 12). However, the clinical use of TNF-α inhibitors and tocilizumab is limited by their high cost and increased risk of infection. Notably, TNF-α inhibitors may also lead to the reactivation of latent tuberculosis. Cyclophosphamide, although immunosuppressive, has a relatively delayed onset of action, requiring several weeks to months to exert its therapeutic effects. Cyclosporine primarily acts by inhibiting T-cell activation and proliferation but has minimal impact on macrophages. Studies by Péral (13) and Paquet (14, 15) have demonstrated that macrophages and their derived myeloperoxidase (MPO) play a critical role in TEN pathogenesis, which are not suppressed by cyclosporine. While the aforementioned agents each have their limitations, our patient’s rapid disease progression and concurrent tuberculosis necessitated alternative therapeutic options with superior efficacy and safety profiles.

To explore novel therapeutic approaches, we systematically reviewed the underlying immunopathological mechanisms of ICI-induced TEN, which primarily involve: 1) Heightened T-cell activity directed against antigens shared by tumor and normal tissues, increased production of autoantibodies, and elevated levels of pro-inflammatory cytokines leading to aberrant immune activation (16); 2) Macrophage infiltration in lesional skin with high expression of TNF and CXCL10, which recruits CXCR3+ cytotoxic T lymphocytes (CTLs) to skin lesions, resulting in epidermal detachment and necrosis. TNF signaling through the NF-κB pathway serving as the core mechanism for CXCL10 production and CTL activation (17). ICI-induced TEN shares remarkably similar immunopathological mechanisms with autoimmune disorders.

Vincristine (VCR), as an immunosuppressant, binds to tubulin, inhibits microtubule polymerization, and blocks cell mitosis, exerting immunosuppressive effects through multiple mechanisms. First, it disrupts immune cell migration and signaling: the microtubule network serves as the structural framework for immune cell motility and intracellular signal transduction. VCR compromises microtubule integrity, inhibiting the chemotaxis and tissue infiltration of macrophages and lymphocytes. In addition, it suppresses the nuclear translocation of transcription factors, such as NF-κB, leading to reduced expression of pro-inflammatory genes, such as TNF-α and interleukin-6 (18). Second, VCR inhibits lymphocyte proliferation and activation: by interfering with microtubule dynamics, it disrupts mitosis in both T and B lymphocytes, effectively preventing their clonal expansion. These findings emphasize VCR’s potent immunosuppressive properties, supporting its potential role as a therapeutic agent for controlling immune hyperactivation induced by ICIs (19).

Accumulating evidence supports that vincristine-containing regimens can enhance therapeutic efficacy in autoimmune diseases. A retrospective study by Stirnemann et al. (20) analyzed data from patients with persistent, or chronic ITP treated with VCR and found that it improved treatment efficacy while maintaining a favorable safety profile. Approximately 20 – 30% of ITP patients are refractory to both glucocorticoid therapy and splenectomy, a condition referred to as refractory ITP. In a study by Jin Jieping et al, patients with refractory ITP were treated with a combination of immunoglobulin and VCR, resulting in favorable outcomes (21). Wu et al. (22) reported that, in patients with systemic lupus erythematosus, sequential therapy with VCR followed by cyclophosphamide demonstrated superior efficacy and a better safety profile compared to high-dose cyclophosphamide monotherapy. Min Hanyi et al. compared the efficacy of high-dose prednisone monotherapy with a cyclophosphamide–VCR–prednisone (COP) regimen in patients with thyroid-associated ophthalmopathy (TAO). Their findings demonstrated superior efficacy of the COP regimen, suggesting it may be more appropriate for patients with highly active or severe TAO (23). Lymphocyte proliferation and CTL activation are closely associated with the pathogenesis of graft-versus-host disease (GVHD). In an *in vitro* study, Lanfranchi reported that treatment with VCR combined with methylprednisolone significantly inhibited peripheral blood lymphocyte proliferation and significantly impaired the generation of functional CTLs (24). At the 2021 American Society of Clinical Oncology meeting, Zhan-Hong Chen et al. reported the use of a VCR–immunoglobulin–glucocorticoid (VIG) regimen to manage grade 3/4 or steroid-refractory irAEs secondary to PD - 1 monoclonal antibody therapy. Treated irAEs included pneumonitis, immune-mediated hepatotoxicity, rash/SJS/TEN, encephalitis, ITP, near-blindness, and severe oral mucositis. The VIG regimen achieved an 88% clinical improvement rate (25).

Given vincristine’s (VCR) established therapeutic efficacy in other autoimmune diseases and its theoretical capacity - disrupt TNF signaling pathways thereby inhibiting macrophage/T-cell activation/migration, reducing inflammatory cytokine production, and ultimately suppressing ICI-induced hyperimmune responses - it represents a promising candidate for clinical application. Our patient was successfully discharged following rapid identification of TEN and prompt escalation of therapy with the VCR, which effectively suppressed the systemic inflammatory response after suboptimal results from first-line glucocorticoid and immunoglobulin therapy. In our case, the total length of hospital stay was only 14 days, suggesting that VCR may significantly reduce the treatment duration of TEN. Our study contributes further evidence supporting the potential utility of VCR in the management of severe irAEs, such as TEN.

## Conclusion

Our study emphasizes the effectiveness of VCR in the management of severe irAEs, such as TEN. This successful case establishes vincristine (VCR) as a clinically viable option for severe irAEs, offering a crucial supplemental approach for cases refractory to conventional treatments with corticosteroids and intravenous immunoglobulins. However, considering the limited data, further studies are needed to validate the safety and efficacy of VCR in broader patient populations. Future prospective studies are essential to refine treatment strategies and improve clinical outcomes in patients with severe irAEs.

## Data Availability

The original contributions presented in the study are included in the article/supplementary material. Further inquiries can be directed to the corresponding author.
